# SERS-Based Optical Nanobiosensors for the Detection of Alzheimer’s Disease

**DOI:** 10.3390/bios13090880

**Published:** 2023-09-11

**Authors:** Feng Gao, Fang Li, Jianhao Wang, Hang Yu, Xiang Li, Hongyu Chen, Jiabei Wang, Dongdong Qin, Yiyi Li, Songyan Liu, Xi Zhang, Zhi-Hao Wang

**Affiliations:** 1 Department of Neurology, Renmin Hospital of Wuhan University, Wuhan 430060, China; 2023103020025@whu.edu.cn (F.G.); 2021283020152@whu.edu.cn (F.L.); 2018305230019@whu.edu.cn (J.W.); yuhang96@whu.edu.cn (H.Y.); 2022203020032@whu.edu.cn (X.L.); 2017302180135@whu.edu.cn (H.C.); 2018305230001@whu.edu.cn (J.W.); qindong@whu.edu.cn (D.Q.); better_double@whu.edu.cn (Y.L.); songyan_liu.med@whu.edu.cn (S.L.); 2018305231125@whu.edu.cn (X.Z.); 2 Center for Neurodegenerative Disease Research, Renmin Hospital of Wuhan University, Wuhan 430060, China

**Keywords:** Alzheimer’s disease, SERS, nanoparticles, fluid biomarkers, disease diagnosis, biosensor

## Abstract

Alzheimer’s disease (AD) is a leading cause of dementia, impacting millions worldwide. However, its complex neuropathologic features and heterogeneous pathophysiology present significant challenges for diagnosis and treatment. To address the urgent need for early AD diagnosis, this review focuses on surface-enhanced Raman scattering (SERS)-based biosensors, leveraging the excellent optical properties of nanomaterials to enhance detection performance. These highly sensitive and noninvasive biosensors offer opportunities for biomarker-driven clinical diagnostics and precision medicine. The review highlights various types of SERS-based biosensors targeting AD biomarkers, discussing their potential applications and contributions to AD diagnosis. Specific details about nanomaterials and targeted AD biomarkers are provided. Furthermore, the future research directions and challenges for improving AD marker detection using SERS sensors are outlined.

## 1. Introduction

**Figure 1:**
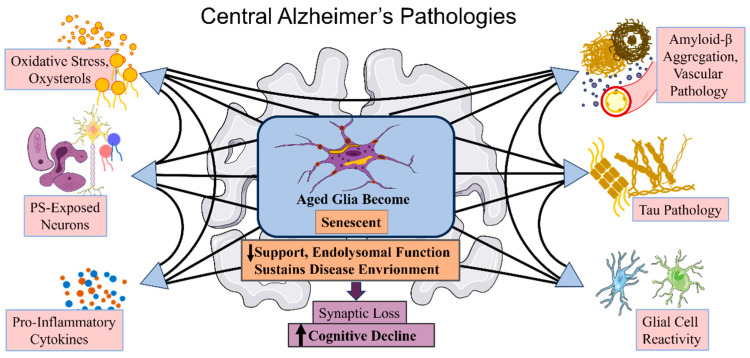
The main pathologies of Alzheimer’s disease ( AD). Risk factors associated with late-onset Alzheimer’s disease (LOAD) are thought to contribute to at least one of six main pathological processes: oxidative stress and oxysterol production, phosphatidylserine-exposed neurons, pro-inflammatory cytokines, amyloid-beta aggregation and vascular pathology, tau pathology, and glial cell reactivity.

Alzheimer’s disease (AD) is a progressive neurodegenerative disorder that poses a serious threat to the health of middle-aged and elderly populations worldwide and is characterized by a progressive decline in two key cognitive functions: thinking and memory impairment [1,2,3]. As the most predominant cause of dementia, AD has been designated as a public health priority disease by the World Health Organization [4]. Based on the epidemiological investigation, it is estimated that more than 150 million people worldwide will suffer from AD by 2050, resulting in 115.8 million disability-adjusted life-years [5,6]. In individuals, the incidence of older than 65 years is expected to sharply increase, with approximately 50% of those over the age of 85 suffering from AD [7]. With the gradual acceleration of global population aging, the number of potential patients diagnosed with AD is far from precise and exceeds our estimates, which places a heavy burden on socio-economic and healthcare systems worldwide [8,9]. Studies on the pathogenesis of AD have found that a variety of important pathological mechanisms are involved in the development of AD, such as the accumulation of amyloid β (Aβ) proteins, hyperphosphorylation of tau, as well as inflammation, oxidative stress, and neuroglia reactivity (Figure 1) [3,10,11,12,13]. When the accumulation of these AD pathologies reaches a certain level, synaptic loss and neuronal death are triggered, leading to cognitive function decline [3,14]. Despite the tremendous efforts being made to explain the pathological process of AD and to find effective drugs for its treatment, the results have been unsatisfactory. To date, almost all drugs approved by the U.S. Food and Drug Administration (FDA) are aimed at improving the symptoms of AD, as there is a lack of understanding of the primary causes of AD [15]. Therefore, diagnosing preclinical AD is critical for scientific treatment and targeted medical interventions to improve patient morbidity and life expectancy [16].

**Figure 2:**
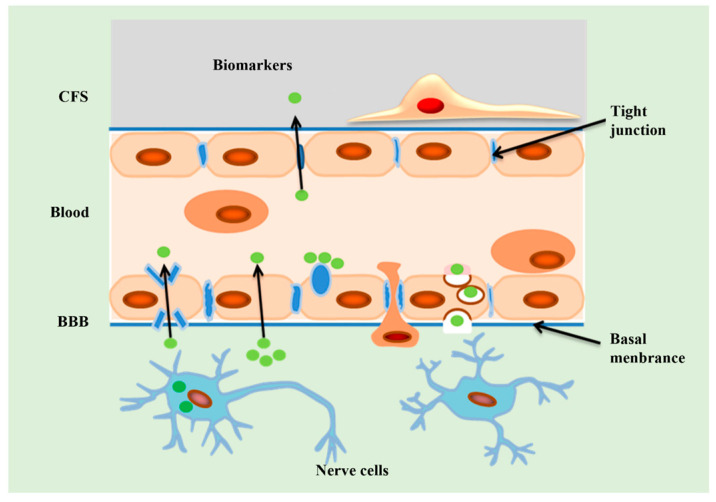
Distribution of AD biomarkers. AD biomarkers can be present intracellular in nerve cells. Additionally, these biomarkers can be present in the bloodstream through various mechanisms, including cellular cytosis, protein channels, and direct osmosis. Furthermore, AD biomarkers can pass through tight junctions and enter the CSF. BBB means blood–brain barrier; CFS means cerebrospinal fluid.

In the current clinical practice, the diagnosis of AD relies on clinical presentation, cognitive screening tools, and medical imaging manifestations [17]. However, there are some limitations in those AD diagnosis methods. For example, an AD diagnosis based on clinical presentation and medical history lacks subjectivity and accuracy due to the high degree of patient heterogeneity [18]; cognitive screening tools are not precise enough to assess cognitive decline [19]; and medical imaging tools, such as magnetic resonance imaging (MRI) and positron emission tomography (PET), are too expensive to be widely used in clinical practice. In addition, radiation further limits their application [20]. Therefore, there is an urgent need for a highly sensitive, easy-to-use, and noninvasive diagnostic method for AD. Disease-related fluid biomarkers have attracted the interest of scientists as non-invasive candidates that can objectively and accurately predict disease progression [21,22]. Based on the distribution of AD markers in the brain (Figure 2), AD biomarker assays used for clinical diagnosis mainly analyze biomarker levels collected from cerebrospinal fluid (CSF) by lumbar puncture or study biomarkers in the brain tissue by MRI or PET [19]. However, these methods are invasive and expensive, prompting researchers to turn to a wider range of serum or plasma biomarkers (Figure 2). The traditional strategies for detecting blood biomarkers include the following techniques: enzyme-linked immunosorbent assay (ELISA) [23], Western blot [24], fluorescence immunoassay [25], electrochemical analysis [26], and polymerase chain reaction (PCR) [27]. Although conventional assay techniques can grossly detect biomarkers, they are not suitable for precise clinical detection because of their complicated procedures, the need for specialized operators, and the low sensitivity.

In recent years, nanomaterial science and spectroscopic detection techniques have been rapidly developing and extending into the field of disease diagnosis due to the unique optical properties of nanoparticles with special shapes and structures and the powerful analytical capabilities of spectroscopic detection techniques. Surface-enhanced Raman scattering (SERS), as a highly sensitive spectroscopic detection technique, has become a hotspot for biological and medical applied research due to its ultra-high sensitivity for non-invasive biomolecular detection, presentation of unique “fingerprint” information in biomolecules, resistance to photobleaching and photodegradation, and low interference in water [28,29]. By employing plasmonic nanomaterials in SERS detection, it is possible to obtain stronger Raman spectra that can increase the sensitivity of the assay. The plasmonic nanostructures of nanomaterial induce the SERS signal enhancement by electromagnetic enhancement produced by the interaction of electromagnetic waves and free electrons in the nanostructures and chemical enhancement producing modified molecules and nanoparticles by electron transfer [30]. Utilizing plasmonic nanoparticle-based SERS methods, fast and sensitive biosensing platforms were designed that show great potential for application in clinical diagnosis. Introducing SERS and nanomaterials into AD clinical diagnosis and optimizing the optical properties of nanomaterials and biorecognition of AD biomarkers by nanomaterials will help to develop point-of-care technologies (POCTs) and screening tools with the advantages of user-friendliness, time-saving, and sensitivity.

This paper reviews various SERS-based nanobiosensors for AD biomarker measurement during the recent years (2011–2023). First, the fabrication process and construction principles of SERS-based nanobiosensor platforms are described in detail, the current status of AD marker research is reviewed, and then, various nanoparticle-based biosensors developed for AD biomarker detection are presented, including label-free nanobiosensors, SERS tag-based nanobiosensors, magnetic separation nanobiosensors, and microfliuid nanobiosensors. Finally, novel SERS-based biosensors are purposely proposed to point out the direction for future AD biomarker research in clinical application studies.

## 2. Principle of SERS

**Figure 3:**
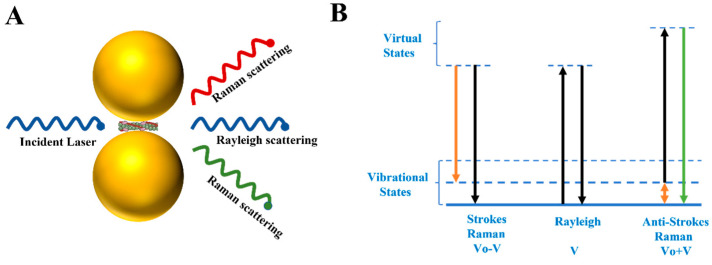
Types of light scattering. (**A**) All three different ways in which light can be re-emitted: Rayleigh scatter, Stokes Raman scatter, anti-Stokes Raman scatter. (**B**) Energy level change for different scattering: the energy level of Rayleigh scatter remains unchanged; the energy level of Stokes Raman scatter decreases; the energy level of anti-Stokes Raman scatter increases.

In 1928, C.V. Raman discovered by chance that during an experiment, incident and scattered light did not have the same frequency when passing through a transparent medium. This interesting phenomenon is now known as Raman scattering [31]. As we know, the incident photons are absorbed by molecules of the medium when passing through it (Figure 3A). In most cases, the molecules would re-emit photons without exchanging energy under laser irradiation (Rayleigh scattering) [32]. However, parts of the re-emitted photons were presented as being higher or lower than the incident photons in terms of energy, and the frequency and direction of the re-emitted photons also changed, which was Raman scattering (Figure 3B). Studies revealed that the intensities of Rayleigh scattering and Raman scattering were reduced to 10^−3^ and 10^−6^ orders of magnitude compared to those of the incident light, respectively [33]. Consequently, there is an urgent need for effective strategies to amplify Raman signal intensity in molecular analysis.

In 1974, Fleischmann and his collaborators discovered for the first time a very special phenomenon: the intensity of the Raman signal of pyridine was enhanced when pyridine was modified on rough silver electrodes [34]. Subsequent studies have demonstrated that Raman signal enhancement also occurred when capturing the Raman spectrum of a molecule attached to the surface of a roughened metal [35]. Later, the concept of surface-enhanced Raman scattering was introduced.

**Figure 4:**
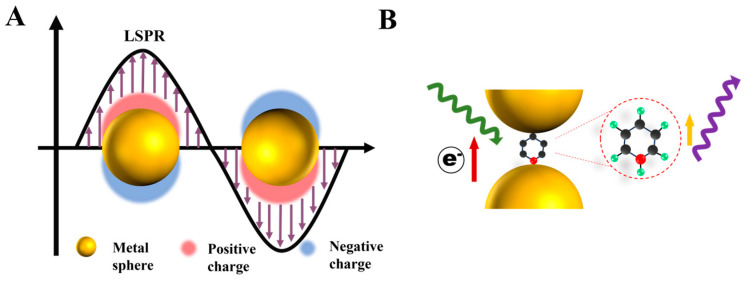
Types of surface-enhanced Raman scattering (SERS) enhancements. (**A**) Electromagnetic enhancement principle of SERS: metal conductive electrons are excited into collective oscillations generating an electromagnetic field highly localized in the metal–dielectric interface when irradiated with light. (**B**) Chemical enhancement principle of SERS: the interaction between nanoparticles and molecules can lead to mutual excitation of the Raman polarizability (indicated by a thin purple arrow) from the local electromagnetic field (indicated by a green arrow), resulting in the generation of enhanced Raman signals from the molecule.

Although SERS is widely used in various fields, the explanation of the theory of SERS enhancement is still not perfect. Currently, the mainstream explanations for SERS enhancement are electromagnetic enhancement and chemical enhancement (Figure 4A) [36]. As the main factor of SERS signal enhancement, electromagnetic enhancement is mainly based on localized surface plasmon resonance (LSPR) [37,38], which is theoretically based on the incident light-induced collective oscillations of free electrons on the surface of plasma nanoparticles. The collective oscillation of free electrons enhances the electromagnetic field intensity around the metallic nanostructure, eventually leading to Raman signal enhancement. The effect of Raman signal enhancement caused by LSPR is roughly proportional to the fourth magnitude of that produced by the metal structure itself. In addition, rough plasmonic nanoparticles can generate inhomogeneous electric field distributions. For instance, the electromagnetic intensity of sharp tips presented on the surface of a rough plasmonic nanoparticle can be sharply enhanced due to the lightning rod effect [39], namely, “hot spots”. The gaps that result from the nanoparticles being in close proximity to each other or overlapping can also create “hot spots”. It is reported that Raman signal amplification caused by “hot spots” is approximately equal to 10^6^~10^8^ power of traditional Raman signal amplification [40].

Another type of SERS enhancement is chemical enhancement (Figure 4B), where the SERS enhancement is caused by electron transfer between the modified molecules and nanoparticles [41]. Notably, the conditions for achieving chemical enhancement require that the distance for electron transfer between the modified molecules and nanoparticles must be less than 10 nm. As a result, the effect of chemical enhancement only reaches two to three orders of the traditional Raman [42]. It is easy to see that chemical enhancement is significantly lower than electromagnetic enhancement in terms of the percentage contribution to the total SERS enhancement. The chemical enhancement can perfectly explain the relationship between the chemical structure of the molecule and its SERS enhancement factor [43].

## 3. SERS-Based Nanobiosensors

Optical biosensors have become widely used tools in biomedical analysis. The design of optical biosensors using SERS nanomaterials has sparked the interest of scientists due to the detection advantages of SERS and the optical properties of nanomaterials. SERS-based nanobiosensors have excellent analytical capabilities and can be perfectly used for biological sample analysis in a non-destructive manner. Thus, biosensors based on SERS reactive nanoparticles have a profound impact on bioassays, especially for disease diagnosis and monitoring.

**Figure 5:**
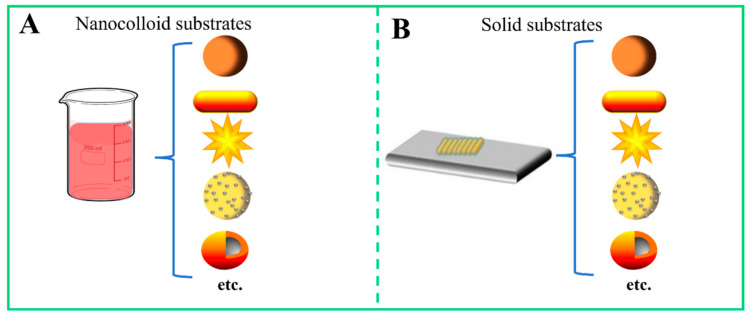
Types of SERS substrates. (**A**) Various nanocolloid substrates, which are constructed by using nanoparticles, nanorods, nanoflowers, alloy nanoparticles, core-shell nanoparticles, and so on. (**B**) Various solid substrates, which are constructed by using nanoparticles, nanorods, nanoflowers, alloy nanoparticles, core-shell nanoparticles, and so on.

As an important component of SERS biosensors, the construct of the SERS substrate is significantly important for performing a SERS experiment, because the enhancing substrate can significantly improve the analysis performance of the biosensor, such as by being sensitive, stable, accurate, reproducible, and so on [44]. Generally, SERS substrates consist of two forms: nanocolloid substrates and solid-based nanostructure substrates [45,46]. Nanocolloid substrates mean that SERS-active nanoparticles were dispersed in the solution when SERS detection was conducted (Figure 5A). The preparation of nanocolloid substrates is much simpler with respect to the solid SERS substrate, with the ability to ignore the interference of water signals and inhomogeneous signals captured from SERS measurement using SERS substrate. However, liquid substrates have many limitations, including difficult long-term storage because of the easy aggregation of nanoparticle colloids, lower SERS activity, and unstable signals in terms of the inherent instability of nanoparticles [47]. According to the report, non-aggregated colloidal substrate only reaches the 10^4^–10^5^ order of magnitude of the SERS analytical enhancement factor (AEF) [48]. Although the fabrication process of solid SERS substrates is relatively complex, solid substrates possess superior analytical performance. The AEF values of solid substrates are in the range of 10^5^ to 10^6^ [48]. The outstanding feature of this special class of SERS substrate is its ability to provide stable and reproducible SERS signals, which is due to its solid features. By optimizing the surface structures of the solid substrate, highly dense and uniform hot spots are obtained, which improve its potential in practical applications [49].

Similarly, SERS active nanomaterials, as the basic element of the SERS substrate, are able to depend on their own special structure and chemical properties to prepare different surface structures of the solid substrate and liquid substrates (Figure 5B). Nanoparticles are one type of nanomaterial, having a size in the nanometer range. The physical and chemical properties of nanoparticles are different from those of bulk materials, whose properties are size-dependent, including optical, magnetic, and electrochemical characteristics, especially those with diameters less than 100 nm [50]. LSPR is an electromagnetic signal amplification mechanism that is strongly influenced by nanoparticle composition, size, shape, dielectric environment, and spacing. By carefully tuning the shape and size of the nanoparticles, the electromagnetic fields at the surface of nanoparticles can be enhanced, resulting in a superior LSPR effect. Based on the electromagnetic signal amplification mechanism, the enhanced vibrational molecular signatures of the target analytes can be measured [51]. Thus, SERS enhancement is mainly based on the non-resonance field enhancement and size-dependent enhancement originated from a competition between SERS enhancement and extinction [52,53]. Many studies have reported that a variety of SERS active nanomaterials are used for the design of SERS substrates, including monometallic nanomaterials of gold, silver, and copper nanoparticles [46,54,55], metal nanocomposites [56], special structure nanomaterials of core-shell nanoparticles [57], carbon materials, metal-organic frameworks [58], and magnetic nanomaterials [59]. By optimizing the size and shape of SERS active nanomaterials, the surface morphology on the SERS substrate, and the combination of the analyst molecules and surface structures of the substrate, the overall enhancement factor (EF) of the SERS substrate can be raised to a higher level [60], resulting in high sensitivity in the detection performance.

## 4. AD Biomarkers

AD is a progressive neurodegenerative disorder, characterized by two major pathological hallmarks: amyloid-beta (Aβ) deposition within senile plaques (extracellular) and the accumulation of hyperphosphorylated and truncated tau protein (intracellular) [61]. According to the pathological features of AD, it is easy to know that Aβ and tau are the two key biomarkers closely related to AD. Aβ is a polypeptide, as well as the initial trigger of AD generated by the metabolism of amyloid precursor protein (APP), which has two common oligomeric subtypes (Aβ_42_ and Aβ_40_) [62]. The initially formed Aβ is protective; however, aggregation and misfolding of Aβ increases its neurotoxicity by mediating reactive oxygen species production, neuroinflammation as well as chronic neuroinflammatory responses, and impaired synaptic function [63]. Additionally, Aβ plaques, as well as smaller accumulations of the Aβ oligomeric form, are reported to be able to interact with metabotropic glutamate receptor 5 and the N-methyl-D-aspartate receptors and other toxin receptors, leading to damage of the normal function of neurons [64]. Moreover, beta-secretase 1 (BACE1), as an important enzyme for amyloid-beta precursor protein cleaving, effectively promotes Aβ oligomeric form production; therefore, BACE1 is considered as another candidate biomarker for AD [65]. Tau is a primary component of microtubules that has a function in stabilizing microtubule assembly [66]. The formation of the tau protein undergoes several modifications after being translated, which serve to regulate its interactions with microtubules. Among those post-translational modification for tau, phosphorylation is the most widely studied. Under pathological conditions, the disruption of balance between tau phosphorylation and dephosphorylation contributes to enhance the capacity of tau to accumulate in the cytoplasm to form paired helical filaments (PHFs), resulting in the collapse of the neuroaxonal protein-binding microtubule and the destruction of neuronal plasticity [67]. In addition, misfolded tau protein by post-translational modification has the ability to cause pathological propagation between cells, similar to prion disease. The tau protein is secreted into the extracellular space as a result of synaptic activity and, subsequently, is taken up by both postsynaptic neurons and glial cells, resulting in neuroinflammatory responses and neuronal death [68]. Therefore, total tau (t-tau) and the phosphorylation of tau (p-tau), including the subtypes of p-tau (p-tau 181, 199, 217, 231), are the predictors for AD [69]. Similar to tau, neurofilaments (NFs) are the fundamental units of the neuron axon with the function to maintain the normal nerve impulse conduction and the structural integrity of neurons. The level of NFs changes when the loss of neurons happens in AD [70].

Cognitive dysfunction is the most prominent clinical manifestation of AD. The generation of cognitive activities, such as memory and thinking, mainly depends on the synaptic activities. The biomarkers related to the synaptic disorders in AD are also regarded as AD biomarkers. For example, neurogranin is a synaptic protein whose concentration variation can reflect the synaptic disorders in AD [71]. Visinin-like protein-1 (VILIP-1) is a protein that regulates synaptic plasticity and takes part in neuronal signaling pathways. In turn, the loss of synapses in AD is able be predicted by VILIP-1 [72].

Numerous studies have shown that inflammation also holds a pivotal role in the pathogenesis and progression of AD [73,74]. Neuroinflammation is characterized by microglia and astrocyte activation and peripheral immune cell aggregation in the brain. Inflammatory factors produced in the course of neuroinflammation seriously impact neuronal function. Immune signaling pathways activating are reported to induce behavioral alterations and cognitive dysfunction [75]. Thus, some biomolecules produced by neuroinflammation can also be used as AD biomarkers. The triggering receptor expressed on myeloid cells 2 (TREM2), as a transmembrane glycoprotein generated by microglia, is highly correlated with tau and neuroinflammation. The research found that the overexpression of TREM2 is able to rescue neuronal and synaptic loss, as well as inhibit tau hyperphosphorylation, further improving AD neuropathology [76]. Glial fibrillary acidic protein (GFAP) is a special cytoskeleton protein for mature astrocytes, having the function of consisting of mature astrocyte structure and movement [77]. In AD neuroinflammatory changes, astrocytes are activated to form reactive astrocytes, which overexpress GFAP in areas surrounding Aβ plaques [78]. Meanwhile, overexpressing GFAP can accelerate tau accumulation in the AD brain. Another protein, chitinase 3-like 1 (CHI3L1/YKL-40), secreted by inflammation-related microglia, is also relatively associated with AD progression [79]. Some studies demonstrated that the level of YKL-40 in the CSF of AD patients is higher than that of normal people. In contrast, the level of YKL-40 in the serum is decreased in AD compared to the control [80].

Cerebrovascular diseases play a key role in promoting AD pathology. Microvascular brain injury (μVBI) alters cerebral blood flow in the brain, resulting in the dysregulation of the microcirculatory function of the brain, such as inadequate oxygen supply and nutritional deficiencies for the central nervous system, further leading to the onset of inflammation, oxidative stress, and nitric oxide disorder in the brain, which are important contributors to AD [81]. It was reported that the concentration of high-heart-type fatty acid-binding protein (hFABP), associated with vascular dysregulation, increased with AD onset [82]. Additionally, the clinical trials revealed that the accuracy of predicting and differentiating against AD increased when using the biomarkers hFABP and p-Tau together [83].

Biomarkers related to heredity for AD have also been gaining attention from scholars. According to the epigenetic study, the occurrence and development of AD are determined by the combined effects of genetic and environmental factors. The research reported that 70% of the risks for AD are correlated with genetics [84]. As a result, gene biomarkers are promising candidates for AD diagnosis. In familial AD and early-onset AD, amyloid-beta precursor protein (APP), presenilin 1 (PS1), and presenilin 2 (PS2) are the most relevant gene biomarkers for AD. Some mutations in those genes cause the production of more toxic Aβ fragments, which play a part in AD onset [85]. Additionally, the mutations of other genes, such as apolipoprotein E (ApoE), bridging integrator1 (BIN1), and cluster protein (CLU), affect the clearance rate of Aβ and increase the accumulation of Aβ, especially in late-onset AD, which possesses a risk of AD [86]. In addition to DNA-related biomarkers, there is another type of heredity-related biomarker called RNA-related biomarkers. RNAs are essential for protein synthesis processes, which are mainly involved in protein transcription and translation processes [87]. Among multiplied RNAs, microRNAs, as small non-coding RNAs, are able to bind with messenger RNA to regulate the expression of related genes. Similarly, variations in the expression of related genes can reflect changes in the synthesis of downstream proteins [88]. Therefore, in theory, miRNAs related to the protein-related AD biomarkers are theoretically expected to be AD RNA-related biomarkers.

Lipids are an essential part of cell membranes and are abundant in the brain, accounting for 60% of the non-aqueous portion [89]. Several genes, such as ApoE, were suggested to be involved in the pathological process of AD [89]. ApoE is a crucial lipoprotein found in the blood that plays a vital role in controlling lipid metabolism, redirecting cholesterol transport, and facilitating their distribution through ApoE receptors and proteins involved in lipid transfer. Furthermore, ApoE is involved in facilitating the exchange of metabolites between neurons and glial cells, promoting synaptogenesis, maintaining neuronal plasticity, remodeling of membranes, modulating immune responses, and aiding in the clearance of Aβ [90]. Therefore, changes in lipids may also play an important role in AD pathology. Some of the research reported that fatty acids, sphingolipids, glycerophospholipids, and various lipid peroxidation compounds were found in CSF and blood at early AD stages [91]. It was reported that the abnormal metabolism of sphingolipids could contribute to AD. Ceramide, as a product of sphingolipid metabolism, was shown to promote the production of Aβ by stabilizing BACE1 and extending its half-life, thereby contributing to a harmful cycle. Similarly, it can also promote AD occurrence through effects on neurofibrillary tangle pathology [92]. Fatty acids and glycerophospholipids are the primary components involved in the formation of cell membranes and have a significant impact on various complex cellular processes, including proliferation, trafficking, and the modulation of membrane proteins and their functions. The dysregulation of these components can activate excitotoxic and inflammatory pathways, impair synaptic function, and contribute to neuronal loss [93]. However, the underlying mechanism of those indicators functioning in AD pathology is not fully understood. Additional studies are needed to investigate the detailed function of lipid-related biomarkers in AD.

In addition to the typical markers mentioned above, there are many other clinically well-studied biomarkers (Table 1) and miRNA-related potential markers (Table 2) for AD due to its complex pathological mechanism. AD biomarkers constitute the alterations in physiology, biochemistry, and anatomy of AD that reveal the distinctive traits of AD-related pathological variations [94]. The liquid biopsy of AD biomarkers is important for the diagnosis and monitoring of AD.

## 5. Application of SERS-Based Nanobiosensors in AD Biomarker Detections

SERS-based biosensors have the advantages of high sensitivity, high specificity, rapid response, easy operation, and real-time monitoring capability, which make them a promising method for biomarker detection. In addition, SERS biosensors have the outstanding advantage of providing rich fingerprint information for target analytes such as proteins, nucleic acids, and lipids, and so on [98]. Therefore, the strategy of using SERS to analyze AD biomarkers has great potential for application in AD clinical diagnosis.

### 5.1. Lable Free Nanobiosensors

**Figure 6:**
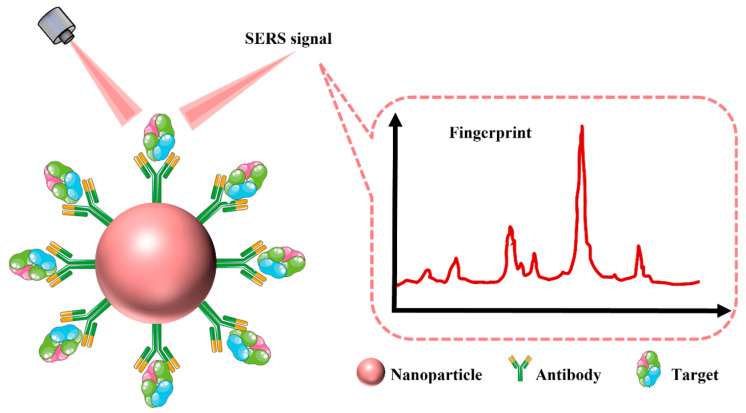
Schematic illustration of the principle of the label-free assay strategy. The fingerprint information for a target can be obtained by detecting SERS spectroscopy, where target molecules are directly attached to the surface of metal nanoparticles without any labels under higher scattering efficiencies.

SERS-based nanobiosensors use a label-free assay strategy to directly analyze target molecules by measuring their intrinsic vibrational fingerprints when modified on the biosensor surface (Figure 6). Label-free biosensors offer several advantages, including providing real-time detection of molecules in a non-invasive manner, saving time in obtaining results in terms of simple testing operation procedures, and being free from interference from tags or labels [30]. These features make them valuable for various applications, such as in the detection of biomarkers in biological samples. In label-free biosensors, Ag and Au are commonly used as SERS-active nanoparticle for constructing the SERS substrate. These nanoparticles have excellent plasmonic properties that enhance the Raman signal and allow for the sensitive detection of target molecules. For instance, El-Said et al. developed a label-free biosensor based on Au nanoparticles for the detection of Aβ_1–40_ peptide. The authors used a sequential modification method, which involved constructing an AuNPs array and immobilizing the corresponding antibodies on the indium tin oxide (ITO) substrate, to create a SERS biosensor. The biosensor demonstrated a strong correlation between the intensity of the SERS signals and the Aβ antigen concentration, indicating its potential use for the accurate and sensitive detection of Aβ_1–40_ peptide levels [99]. Yan et al. took advantage of the Aβ_40_ monomer and fibrils to synthesize AuNPs in situ. This novel approach can achieve the real-time monitoring of the Aβ aggregation process [100]. Similarly, Kazushige et al. used the gold colloid with an 8 nm diameter for Aβ_1–40_ or Aβ_1–42_ in-situ detection at different pH. It was found that gold colloid-coated Aβ_1–40_ or Aβ_1–42_ was prone to aggregate in an acidic environment (pH~4) in the AD mouse model. This study provided a new strategy based on SERS for amyloid fibril detection in vitro [101]. In another study, Yang et al. utilized a 200 nm thickness of gold granular film to construct the SERS substrate for the investigation of the aggregation behavior of β-amyloid peptides. The result suggested that the metal ions contribute to the aggregation of Aβ_42_ [102]. Unlike the above-mentioned researchers, Buividas et al. employed AuNPs as the fundamental element for developing the SERS substrate, but they also provided a novel method for fabricating the SERS substrate. The unique SERS substrate with grating-like ripple patterns was created utilizing femtosecond laser technology and magnetron sputtering to cover it with AuNPs. Meanwhile, the developed SERS substrate exhibited a high sensitivity for low Aβ_40_ oligomer concentration detection in the range from 10 nM to 10 µM [103]. In addition to gold nanoparticles, silver nanoparticles also have strong SERS signal enhancement owing to their high plasmonic activity. For example, Hao et al. employed an Ag film substrate modified with the spacer molecule IDA to explore tau phosphorylation in situ. The SERS substrate was demonstrated to have good SERS spectral reproducibility. More importantly, it had great capability in discriminating TauS214 from TauS396, suggesting good potential for clinical applications [104]. As for constructing special SERS substrates, composite nanoparticles have been reported to have advantages over single nanoparticles. This is because composite metal nanoparticles incorporate the advantages of all single metal nanoparticles and show a stronger surface plasmon resonance (SPR) effect [105]. Wang et al. used platinum (Pt) as the foundation framework to fabricate l/d-Pt@Au triangular nanorings. This kind of composite metal nanoparticle-based SERS substrate can make full use of the surface-enhanced Raman scattering-chiral anisotropy (SERS-ChA) effect to improve SERS activity. The experimental results showed that the developed SERS biosensor was sensitive in detecting Aβ_42_ with a limit of detection (LOD) of 4 × 10^−15^ M [106]. Prucek et al. created a strep @mPS-AuNPs composite for detecting tau. Dopamine was used to reduce gold and silver ions on the surface of magnetic polystyrene beads to generate the bulge that produces the SERS signal enhancement. The composite’s lower magnetic properties reduce the tendency for sedimentation and increase the SERS detection reproducibility. The SERS substrate was shown to have enormous potential for the detection of biomarkers in intricate biological samples [107]. Graphene has good electron transport and mechanical characteristics, making it a valuable material for SERS substrates, exhibiting high sensitivity, reproducibility, and stability, as well as the capacity to detect at the micron level. Thereby, the graphene is extensive used for the fabrication of composite nanomaterials. Yu et al. constructed a novel graphene–plasmonic hybrid platform and achieved the ultra-sensitive detection of Aβ. The platform is a kind of two-dimensional substrate consisting of two layers (a graphene layer and a nanopyramid array layer). The graphene layer not only provides localized hotspots for precise positioning but also enhances the SERS signal by charge transfer between the analyte and graphene [108].

**Figure 7:**
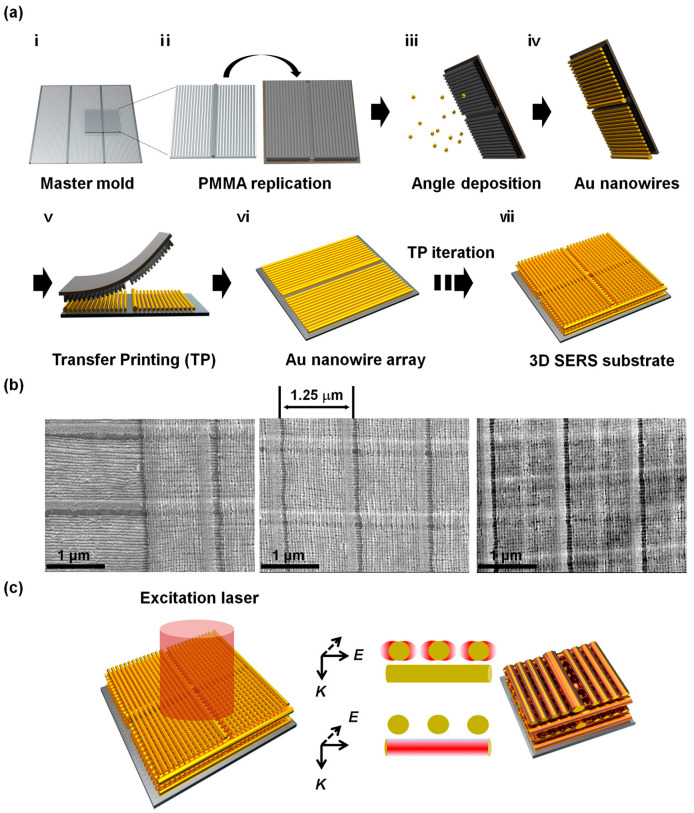
Schematic illustration of a 3D SERS substrate via solvent-assisted nanotransfer printing. (**a**) Schematic of the S-nTP process to form a 3D SERS substrate.i means the master mold pattern; ii means PMMA replication via spin-coating method; iii and iv mean gold nanowires were formed through angle deposition of gold via e-beam evaporation on the PMMA replicas; v means the adhesion between the PMMA and the PI film; vi means a single layer of gold nanowires was successfully transfer-printed on the substrate by gentle pressing with a PDMS pad; vii means 3D SERS substrate is successfully constructed. (**b**) SEM images of the 3D SERS substrate consisting of stacked gold nanowire array sheets. A region where the first and the second sheet overlaps (**left**). SEM images of the 3D SERS structure stacked with two sheets (**middle**) and four sheets (**right**). (**c**) Illustration of the 3D SERS substrate illuminated with a Raman excitation laser and strong local E-fields formed around nanowires according to the excitation laser polarization. The figure adapted from ref. [109] Copyright © 2020 American Chemical Society.

Additionally, a graphene-based three-dimensional SERS substrate was designed by Hyung et al. for label-free assays of the tau protein and amyloid β. The gold nanowire arrays were uniformly deposited in the adjacent voids of graphite nanospheres by a facile solvent-assisted nanotransfer-printing method, and then, the constructed substrate was functionalized with the carboxylic acid (Figure 7). The novel, carboxylic acid-functionalized, graphitic nanolayer-coated 3D SERS substrate (CGSS) features woodpile-type 3D plasmonic structures with highly regular arrays of fine and dense gold nanowires. The special nanostructure substrate is able to overcome the inhomogeneous distribution of the SERS signal on the SERS active substrate by effectively immobilizing the protein to avoid the coffee-ring effect, showing a high enhancement factor (EF) of 5.5 × 10^5^. More importantly, the CGSS has the ability to be employed in secondary structure analysis and the quantitative detection of proteins, which is difficult for other biosensors [109].

### 5.2. SERS Tags-Based Nanobiosensors

**Figure 8:**
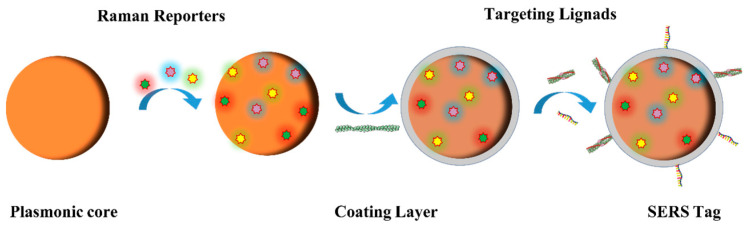
Schematic illustration of the tag synthesis process. First, the plasmonic core is modified with Raman reporters and then coated with a protective layer such as bovine serum proteins, which are used for enclosing nonspecific binding sites. Finally, targeting ligands, such as antibodies and aptamers, are attached to the surface of the plasmonic core to form SERS tags.

The label-free SERS analysis approach for biomarkers provides rapid and direct information about the marker molecule itself. However, it may not be suitable for analyzing complex biological systems. Additionally, the direct SERS analysis method is not sensitive enough for analytes with small Raman cross-sections. These limitations can be overcome by using the SERS tag detection approach. SERS tags are composed of Raman reporters with larger cross-sections and SERS-active nanomaterials. When functionalized with bioligands and covered with a protective shell, SERS tags can specifically capture target molecules and remain stable in complex biological samples (Figure 8). The Raman-silent region (1800–2800 cm^−1^) is a challenging area to obtain Raman signals from endogenous biomolecules. SERS tags can be used to generate distinct Raman characteristic peaks during biomolecule spectrum capture, particularly in the Raman-silent region. This makes SERS tag-detection technology an effective means of achieving specific identification and quantitative analysis of Alzheimer’s disease biomarkers.

**Figure 9:**
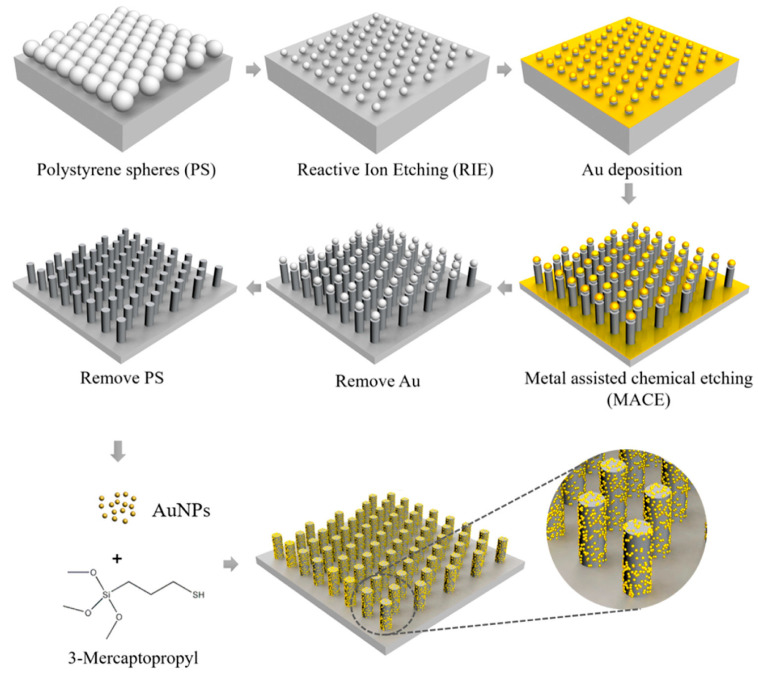
Schematic illustration of the fabrication of AuNP-conjugated SiNR arrays. Close-packed monolayer of PS nanospheres on a clean Si-reduced diameter of PS by reactive ion etching, Au deposition, metal-assisted chemical etching, removal of Au/PS, conjugation with Au nanoparticles. The figure is adapted from ref. [118] Copyright © 2017 American Chemical Society.

For instance, Xia et al. developed a SERS tag, which is AuNPs conjugated with the distinctive spectrum of rose Bengal, to address the problem of difficulty detecting characteristic Raman fingerprints in proteins with intrinsic complex conformations. Furthermore, the RB-AuNPs tag was successfully applied in Aβ_42_ peptide SERS detection [110]. Luca et al. designed a SERS tag for SERS detection of amyloid oligomers by decorating the functionalization of AuNPs with polystyrene beads. The Al^3+^ ions chelated in the functionalization of AuNPs can provide effective adsorption sites for binding misfolded oligomers. The quantitative detection of toxic protein oligomers is made by mechanical deformations of the phenyl ring [111]. Another study reported that Zhang et al. used the oligonucleotide as the aptamer for conjugating with the target protein and employed different exogenous dyes with unique Raman fingerprints as Raman reporters to develop a novel SERS tag. This type of SERS tag can shorten the assay time and is relatively easy to produce. More importantly, it can be used for the multiplexed detection of biomarkers [112]. Credi et al. further optimized the carrier of the SERS tags. They assembled the SERS tag into optical fibers to form cap-shaped SERS sensors. The novel optical sensor was demonstrated to be modulated in its resonant properties range from 520 nm to 800 nm and successfully achieved the specific detection of Aβ peptides [113]. Silver nanoparticles are also commonly used to synthesize SERS tags. Choi et al. modified the Raman reporter of 4-MBPA on the Ag substrate surface for the detection of dopamine, which is an important biomarker for AD. The SERS tag shows high sensitivity and selectivity with a LOD up to 1 pM [114]. Similarly, Zhu et al. attached the Raman reporter of DSNB to an Ag nanoparticle film to construct the Ag-DSNB SERS tag. The reliable sensor can rely on subtle electronic changes during the conjugation between the Raman reporter and Aβ_42_ to in situ monitor Aβ_42_ aggregation and detect the Aβ_42_ monomer. The results suggested the sensor has a LOD of ~10^−9^ mol/L [115]. Jaiswal et al. employed the electrospun nanofibrous mat as a carrier platform and modified it step by step with AgNPs and Rhodamine-6G dye. The LOD of the flexible SERS sensor was found to be 76 × 10^−18^ M during the measurement of Aβ_1–4_ [116]. Jin et al. created a special SERS tag based on a silver nanogap shell (AgNGSs) structure. They synthesized the AgNGSs-based SERS tags with different size nanogaps by regulating reaction kinetics using different Raman labels. As we know, proper nanogaps are able to produce many plasmonic “hot spots”, resulting in the dramatic amplification of the target analytes’ SERS signal. Accordingly, this SERS sensor presented a lower LOD, less than 0.25 pg mL^−1^, in the detection of Aβ_40_ and Aβ_42_ [117]. Composite nanomaterials are also very competitive in SERS tag preparation due to their additional properties. Lin et al. reported AuNPs-functionalized Si NR arrays modified with R6G as the SERS tag (Figure 9). By uniformly distributing the AuNPs on the hexagonal-packed Si nanorod (Si NR) arrays, the SERS substrate can form lots of uniform hot spots and improve SERS detection reproducibility. Additionally, the ordered Si-based SERS substrates are able to couple and stabilize hot spots due to the interconnection between hot spots and the semiconducting Si substrates. Furthermore, it was suggested that large molecules such as amyloid fibrils can also be detected by tuning the diameter and gap between neighboring rods. It was demonstrated that the SERS sensor can offer highly sensitive, uniform, and reproducible SERS signals in the detection of a single-Aβ fibril level with a small RSD of ~3.9–7.2% [118].

The SERS-tag-based double antibody sandwich assay is an ELISA-like analysis method that consists of two parts: the SERS probe and the SERS substrate. This special structure of the immune complex can significantly improve the specificity of antigen-antibody recognition and produce a strong Raman signal enhancement effect by pulling Raman-active nanoparticles closer together to generate more “hot spots”. Yang et al. used half-antibody fragments to fabricate SERS nanotags and designed a head-flocked nanopillar substrate. The analytes are connected to both the SERS tag and the nanopillar substrate in a sandwich structure. This sandwich structure was demonstrated to decrease the distance between the nanopillar substrate and the SERS tag by half due to the length of the half antibody fragment. As a consequence, a large number of plasmonic couplings formed in the sandwich structure, leading to a 135-fold increase in detection sensitivity over a SERS sensor based on whole antibodies. As for tau protein detection, the developed sensor has a wide detection range from 10 fM to 1 μM and a LOD of up to 3.21 fM [119]. In another study, Adem et al. proposed the use of a hybrid magnetic nanoparticle modified monoclonal antitau as the probe and polyclonal antitau and 5,5-dithiobis (2-dinitrobenzoic acid) functionalized AuNPs as SERS tags for tau specificity detection. Tau was sandwiched with SERS probes and SRES tags to form aggregate structures, producing a SERS intensity enhancement. Compared to the current methods, the advantages of the SERS sensor are the fast detection speed (less than 1 min) and the simple preparation process, as well as a lower LOD below 25 fM [120].

### 5.3. Magnetic Separation-Nanobiosensors

**Figure 10:**
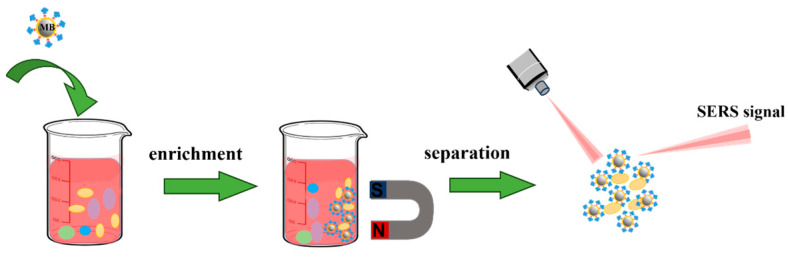
Mechanism of the magnetic separation-SERS based detection. First, magnetic nanoparticles are added into the sample to capture target molecules. and then, the captured complexes are separated and enriched using a magnetic device. Finally, the separated complexes are detected by the SERS detection device. MB means magnetic bead.

Tandem SERS methods other than conventional SERS-based methods can achieve a high sensitivity analysis of target molecules in complex sample systems by various advanced separation techniques. The target molecules were recognized and separated by the separation device, then, deposited on the detection device for SERS analysis. Using the “two-step” strategy, the target analytes are enriched from complex samples by pre-treatments, which is the key point for ultra-sensitive measurement. As for different separation devices, magnetic separation devices are the most classical (Figure 10). Meanwhile, this magnetic separation device is simple to operate, and its principles are easy to understand. In a word, corresponding antibodies or aptamers are modified on the surface of magnetic nanoparticles for capturing the analytes from the samples and separating and enriching the analytes from the samples by magnetic force. The obtained complex can link to or deposit on the SERS substrates for SERS detection. For example, Maurer et al. reported a SERS-based magnetic immunosensor for the purification and detection of the tau protein. In this study, Fe_x_O_y_ nanoparticles were conjugated with polyclonal antitau antibodies as magnetic components for the separation of tau from CSF. Subsequently, AuNPs functionalized with 5,5-dithiobis (2-nitrobenzoic acid) (DTNB) and polyclonal and monoclonal antitau antibodies were coupled with the magnetic components to form the sandwich structure through the tau protein. The construction of SERS-based magnetic immunosensors by a step-by-step method was successfully investigated by different characterization means [121]. Zhang and co-workers developed a dual-mode magnetic immunosensor for blood phosphorylated tau analysis, which combined colorimetric detection and SERS detection. They used antibody-functionalized superparamagnetic iron oxide nanoparticles to capture and separate p-tau_396,404_ from the blood of an AD patient. Furthermore, the AuNPs modified with horseradish peroxidase-labeled antibody R1 and 4-mercaptobenzonitrile (4-MBN) were used as dual-reporters for both colorimetric and SERS signals. When the dual-mode magnetic immunosensor was applied for blood p-tau_396,404_ detection, the LOD for the SERS mode and colorimetric mode were able to reach up to 1.5 pg/mL and 24 pg/mL, respectively [122]. Teresa and her team dispensed the iron magnetic core-gold plasmonic shell nanoparticle into the 2D hybrid graphene oxide to develop a magnetic–plasmonic nanoplatform for SERS “fingerprint” detection of β-amyloid and tau proteins. The iron magnetic core-gold plasmonic shell nanoparticles of this magnetic-plasmonic nanoplatform not only have the ability to enrich the AD biomarkers from whole blood but also provide strong plasmon-coupling originating from the “hot spot” of the core-shell gap. Similarly, the interaction of aromatic molecules existing in the sp^2^-carbon 2D nanosheets and highly electronegative oxygen species anchored on the 2D graphene oxide both contribute to local electric field enhancement via the SERS chemical enhancement [123,124] Consequently, the results showed that the nanoplatform exhibited higher sensitivity for AD biomarker detection than the traditional ELISA kit [125]. Similar to the study of Teresa et al., Yu and colleagues utilized magnetic graphene oxide (Fe_3_O_4_@GOs) as a separation device to isolate AD biomarkers from real serum samples. By encapsulating Ag nanoparticles with tannic acid, they improved the stability of the silver probe, enabling them to maintain a strong Raman signal intensity for up to 30 days. Additionally, they utilized the sandwich assay to specifically capture target proteins and achieved the highly sensitive detection of Aβ_1–42_ (ranging from 100 pg/mL to 10 fg/mL) and p-tau_181_ protein (ranging from 100 pg/mL to 1 fg/mL) [126].

### 5.4. Microfliuid Nanobiosensors

**Figure 11:**
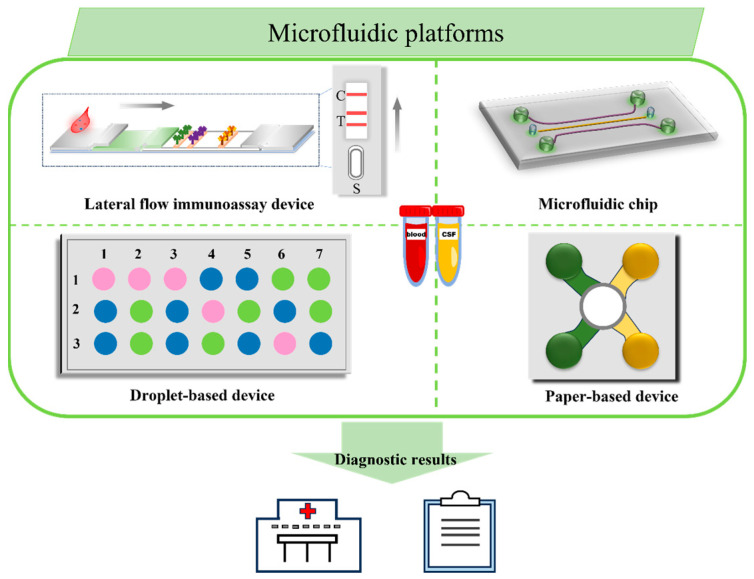
Schematic illustration of multiplied microfluidic platforms. The multiplied microfluidic platforms consist of lateral flow immunoassay devices, microfluidic chips, droplet-based devices, paper-based devices, and so on, which have the advantages of small size and simple operation and can rapidly and accurately diagnose AD by analyzing the blood and CFS from the patients.

Over the past decades, microfluidic technology has experienced rapid development. Microfluidic-based biosensors (Figure 11) have been extensively applied in various fields, including food safety [127], environmental monitoring [128], and clinical medicine [129], which is attributed to its outstanding advantages, such as high throughput, low sample requirement, and multifunctional integration [130]. Microfluidic-based biosensors are known for their relatively small size, which makes them highly portable. In addition, they are capable of precisely controlling the volume of incoming liquid, allowing for semi-automatic or fully automatic quantification while minimizing the interference between different reaction systems, resulting in highly precise and reliable multi-measurements, which are crucial for clinical transformation in terms of disease diagnosis. In our daily lives, the most familiar representative microfluidic sensors are lateral flow immunoassay (LFIA) sensors, which have many advantages other than traditional sensors, including good biocompatibility, ease of patterning, flexibility, portability, high sensitivity, high specificity, and a low cost [131]. Driven by the capillary force, LFIA sensors enable fluid automation detection without equipment or skilled operators. Arranging various test lines in one LFIA device, the LFIA sensors can realize the discrimination of different targets by spatial resolution. Among the various LFIA sensors, the paper-based LFIA sensor is one of the most widely used applications due to its cheap, self-contained capillary effect and facilitation of customization [132]. A SERS-based dual-mode paper-based microfluidic immunosensor was developed by Zhang et al. for p-tau_369,404_ quantitative analysis. In this study, AuNPs functionalized with 4-MBA were modified with the antibody 3G5 to prepare the captured p-tau_369,404_-4-MBA@AuNP-3G5 complex, which is capable of accurately capturing soluble p-tau_369,404_ while also providing strong SERS signals. The 4-MBA@AuNP-3G5 complex capturing P-tau_369,404_ was migrated to the test line by the capillary force and subsequently captured by the paired antibody 4B1 sprayed on the T-line. The author demonstrated that the developed paper-based microfluidic immunosensor possessed high specificity and sensitivity in practical testing, whether in colorimetric assay mode (LOD = 60 pg/mL) or SERS assay mode (LOD = 3.8 pg/mL) [133]. In another paper-based microfluidic immunosensor research project, a gold-core silica shell (Au@SiO_2_) was utilized as a SERS tag to construct multiple detection paper-based microfluidic immunosensors for AD biomarkers. This is because the silica shell has the function of avoiding the dissociation of Raman dyes, thus, improving the stability of detection. The target proteins in the sample were recognized and attached to the surface of different SERS tags (Au^4-MBA^@SiO_2_ and Au^DNTB^@SiO_2_ modified with corresponding antibodies, respectively). Then, the various sandwich hybrid complexes were formed on the designed two test lines on the paper-based microfluidic immunosensor. In practices for the detection of four AD biomarkers (Aβ_42_, Aβ_40_, tau, and NFL), this microfluidic immunosensor exhibited excellent stability and high sensitivity. In the limited detection range of 0.001–1000 ng/mL, the four AD biomarkers were calculated as 138.1, 191.2, 257.1, and 309.1 fg/mL, respectively. The detection time of this microfluidic immunosensor was also reduced to within 30 min [134].

Microfluidics chips, as another type of microfluidic sensor device, are also commonly used for disease detection. Depending on the special properties of polymeric materials for building composite substrates, microfluidic channels in microfluidic chips can be easily prepared by the present processing technologies, such as hot pressing, injection molding, photolithography, and laser etching [127]. Taking advantage of the well-designed microfluidic channels, the multistep continuous reactions of detection can be integrated into a single microfluidic chip and realized automatically in the microfluidic channels. Thereby, the detection performance of microfluidic chips is significantly improved. Sun and their team designed a microfluidic chip based on the polystyrene/Au nanoparticles (PS/Au) composite substrate for the dual detection of Aβ_1–42_ and p-tau_181_ proteins. Making full use of the synergistic coupling between the ability of the PS microsphere to confine light and the LSPR of AuNRs. The PS/Au active SERS substrate exhibited a stronger ‘hot spot’ effect and provided further enhancement of the SERS signal. By constructing a multipath control unit and introducing an external mechanical pump, the microfluidic chip allowed the simultaneous, rapid, and automated detection of Aβ_1–42_ and p-tau_181_ proteins without interfering with each other. The SERS response of the microfluidic chip was lower than 100 fg/mL, suggesting that the device is sensitive enough for the early screening of AD patients at home rather than in hospitals [135]. Unlike the microfluidic biosensor of Sun et al., Chou et al. designed an interesting nanofluidic device. By preparing different nanochannel depths, the gold colloid (60 nm nanoparticles) and Aβ _1–40_ were trapped at the entrance to the nanochannel when they passed through the 40 nm scale of the nanochannel. A high density of gold nanoparticle clusters mixed with the target molecules of Aβ_1–40_ was formed over time, which could provide a high SERS active environment and further improve the detection sensitivity. Furthermore, the mixture was aggregated at the same location as the entrance to the nanochannel when performing the detection, which gives the biosensor high reproducibility. This microfluidic biosensor was demonstrated to have the ability to facilitate the diagnosis and understanding of AD by analyzing the Aβ structure [136].

Droplet-based microfluidic devices are indeed a promising assay strategy that allow for highly parallel and controlled reactions, particularly in applications of high-throughput screening [137]. The droplets act as tiny reaction vessels, enabling reactions to be performed on a large scale while using minimal amounts of reagents. Meanwhile, each tiny reaction droplet as an independent bioreactor can avoid mutual interference while enabling multiplied detection [138]. Unfortunately, to date, there is little research about droplet-based microfluidic devices that is reported in the detection of AD biomarkers. However, the droplet-based microfluidic devices have a lot of potential in AD detection.

## 6. Conclusions and Future Perspectives

Rapid and accurate biomarker assays for AD are essential for screening and diagnosing patients with AD, effectively assessing patient status, and scientifically providing treatment guidelines. Here, we focus on the development and application of SERS biosensors for AD-related biomarker analysis. SERS-based biosensors have great potential as a powerful analytical tool for clinical disease diagnosis and screening. The key to applying SERS-based biosensors for disease diagnosis is to construct ideal SERS substrates and employ different detection modes. Depending on the nature of the sample, the optimal detection mode is selected to obtain the best analytical results. In the future, the introduction of novel SERS-active nanomaterials, the modification of SERS substrates, and the optimization of detection modes will solve this challenge. For instance, when using label-free biosensors for surface-enhanced Raman spectroscopy (SERS) fingerprint analysis of target molecules, it is possible to enhance the detection performance by discovering new materials, such as alloys and organic framework metals, and optimizing their particle size and shape. For SERS labeling-based biosensor detection, the optimized antibodies and novel Raman reporter molecules are good choices. By selecting a shorter antibody, the signal intensity of nanoparticles is enhanced by bringing nanoparticles closer together. Novel Raman reporter molecules should provide better antigen–antibody affinity and possess a larger molecular cross-section, resulting in increased detection sensitivity. More importantly, the construction of reproducible, sensitive, and recyclable SERS substrates is essential for SERS detection, which could be accomplished using modern lithography. Combining magnetic separation strategy and microfluidic chip technology is a good idea in practical application for the low-abundance sample assay. Magnetic separation strategies can not only save costs through the easy recycling of nanomaterial substrates but can also realize low-abundance target enrichment. Meanwhile, microfluidic chip technology is able to provide accurate, fully automated, high-throughput results and quantitative analysis. All of those advantages are important for practical application and promotion. In order to further improve the practical clinical applications, more efforts should be made to establish a more miniaturized, automated, and multifunctional analytical platform for SERS microdevices. Meanwhile, the introduction of biomarkers that can more objectively characterize AD diseases and their combination with SERS-based nanobiosensors to detect AD can realize the assisted diagnosis of AD and improve the sensitivity of diagnostic results. In the near future, with scientists’ further in-depth research on AD pathomechanisms and the development of nanomaterials science, SERS-based nanobiosensors for AD diagnosis and screening will become even more advanced, which will have a great impact on treatment and intervention decisions for AD patients.

## Figures and Tables

**Table 1:** **Cerebrospinal fluid** (CSF) and blood biomarkers for the diagnosis of AD [21,95], which references the database of AlzForum Foundation Inc., Boston, MA, USA (alzbiomarker/ad-vs-ctrl | Alzforum. (n.d.). Henrik Zetterberg, 2016. Retrieved August 1, 2023, from https://www.alzforum.org/alzbiomarker/ad-vs-ctrl).

CSF and Blood Biomarkers for AD	*p* Value	Number of Samples
tau-total (CSF)	*p* < 0.0001	N = 11,596
Aβ42 (CSF)	*p* < 0.0001	N = 10,708
tau-p181 (CSF)	*p* < 0.0001	N = 8808
Aβ42 (Plasma and Serum)	*p* = 0.02998	N = 6020
Aβ40 (CSF)	*p* < 0.0001	N = 2011
Aβ40 (Plasma and Serum)	*p* = 0.15097	N = 5483
tau-total (Plasma and Serum)	*p* < 0.0001	N = 4168
YKL-40 (CSF)	*p* < 0.0001	N = 2070
NFL (CSF)	*p* < 0.0001	N = 1950
Aβ42 (CSF)	*p* < 0.0001	N = 1476
tau-total (CSF)	*p* < 0.0001	N = 1462
albumin ratio (CSF)	*p* = 0.00211	N = 1262
α-synuclein (CSF)	*p* = 0.00038	N = 1140
neurogranin (CSF)	*p* < 0.0001	N = 1389
tau-p181 (CSF)	*p* < 0.0001	N = 1242
tau-p181 (Plasma and Serum)	*p* < 0.0001	N = 1774
Aβ38 (CSF)	*p* = 0.03363	N = 827
sAPPβ (CSF)	*p* = 0.62907	N = 740
NFL (Plasma and Serum)	*p* < 0.0001	N = 1526
sAPPα (CSF)	*p* = 0.43779	N = 559
sTREM2 (CSF)	*p* < 0.0001	N = 686
MCP-1 (CSF)	*p* = 0.00181	N = 723
NSE (CSF)	*p* = 0.00416	N = 211
MCP-1 (Plasma and Serum)	*p* = 0.41128	N = 723
Aβ40 (CSF)	*p* = 0.98641	N = 461
GFAP (Plasma and Serum)	*p* < 0.0001	N = 1123
hFABP (CSF)	*p* < 0.0001	N = 374
VLP-1 (CSF)	*p* < 0.0001	N = 385
GFAP (CSF)	*p* = 0.05530	N = 128
tau-p231 (CSF)	*p* < 0.0001	N = 111
YKL-40 (Plasma and Serum)	*p* = 0.01721	N = 685
Aβ40 (Plasma and Serum)	*p* = 0.61737	N = 557
Aβ42 (Plasma and Serum)	*p* = 0.78533	N = 557
YKL-40 (CSF)	*p* = 0.07985	N = 266
neurogranin (CSF)	*p* = 0.00299	N = 170
hFABP (Plasma and Serum)	*p* = 0.38915	N = 139
NSE (Plasma and Serum)	*p* = 0.99192	N = 97
sAPPβ (Plasma and Serum)	*p* = 0.30006	N = 178
α-synuclein (Plasma and Serum)	*p* = 0.32567	N = 78
MCI-Stable: sAPPα (CSF)	*p* = 0.19510	N = 169
sAPPβ (CSF)	*p* = 0.58568	N = 169
tau-total (Plasma and Serum)	*p* = 0.01547	N = 243
α-synuclein (CSF)	*p* = 0.05789	N = 75
neurogranin (Plasma and Serum)	*p* = 0.25272	N = 49
sAPPα (Plasma and Serum)	*p* = 0.11352	N = 151
tau-p217 (CSF)	*p* < 0.0001	N = 249
tau-p217 (Plasma and Serum)	*p* = 0.08162	N = 393
albumin ratio (CSF/Blood)	*p* = 0.34338	N = 142
Aβ38 (CSF)	*p* = 0.01000	N = 144
VLP-1 (CSF)	*p* = 0.02008	N = 41
p-tau181 (Plasma and Serum)	*p* = 0.0023	N = 157
p-tau217 (Plasma and Serum)	*p*< 0.001	N = 157
p-tau199 (Plasma and Serum)	*p* = 0.0425	N = 157
p-tau202 (Plasma and Serum)	*p* = 0.00164	N = 157
p-tau231 (Plasma and Serum)	*p* = 0.0185	N = 157

**Table 2:** MiRNA-related biomarkers for AD [96,97].

MiRNA Related Pathologies	The Different Function of miRNA	
	Up-Regulated	Down-Regulated
Aβ deposition	miR-149-5p, miR-128, and miR-12	miR-520c, miR-124, miR-101, miR-107, miR-328, miR-29 and miR-29a/b-1, miR-298, miR-16, miR-17, miR-9, miR-195, miR-106, miR-15b, and miR-132-3p
Highly phosphorylated tau protein aggregation	miR-483-5p, miR-181c-5p; miR-125b, miR-26b, miR-199a, miR-34a, miR-146, and miR-146a	miR-106b, miR-15a, miR-101, miR-5m12, and miR-132/-212
Damage to synaptic function	miR-181a, miR-186-5p, miR-26b, miR-30b, miR-124, miR-574, miR-206, miR-142-5p, miR-34a, and miR-199a	miR-10a and miR-188-5p
Neuroinflammation	miR-485-3p, miR-206, miR-32-5p, miR-155, miR-125b, and miR-146a	miR-132, miR-22, miR-331-3p, miR-26a, miR-29a, and miR-let-7a
Autophagy damage	miR-204, miR-214-3p, miR-299-5p, miR-132/212, miR-331-3p, and miR-9-5p	

## Data Availability

Not applicable.
